# Does IQ Really Predict Job Performance?

**DOI:** 10.1080/10888691.2014.983635

**Published:** 2015-01-07

**Authors:** Ken Richardson, Sarah H. Norgate

**Affiliations:** ^a^ Independent Researcher; ^b^ University of Salford

## Abstract

IQ has played a prominent part in developmental and adult psychology for decades. In the absence of a clear theoretical model of internal cognitive functions, however, construct validity for IQ tests has always been difficult to establish. Test validity, therefore, has always been indirect, by correlating individual differences in test scores with what are assumed to be other criteria of intelligence. Job performance has, for several reasons, been one such criterion. Correlations of around 0.5 have been regularly cited as evidence of test validity, and as justification for the use of the tests in developmental studies, in educational and occupational selection and in research programs on sources of individual differences. Here, those correlations are examined together with the quality of the original data and the many corrections needed to arrive at them. It is concluded that considerable caution needs to be exercised in citing such correlations for test validation purposes.

IQ has now been used as a measure of cognitive functioning for over a century. It has played a prominent part in developmental studies in many ways: as an index of normal development; for clinical diagnostics; as a descriptor of individual differences in cognitive ability; as explanation for differences in achievement or success in the world; as a predictor of future success as in school, training and occupational selection; and as an index for exploring causes of individual differences in cognitive ability. For example, it is argued that the current search for associations between molecular genetic variations and IQ “will transform both developmental psychology and developmental psychopathology” (Plomin & Rutter, 1998, p. 1223; see also Plomin, 2013). Likewise, Kovas, Haworth, Dale, and Plomin (2007) say that their conclusions on the heritability of IQ “have far-reaching implications for education and child development as well as molecular genetics and neuroscience” (p. vii). Clearly, a lot hinges on the validity of the test, especially as such studies are very expensive.

The validity of an IQ test—or what it actually measures—on the other hand, has always been a difficult subject. Since Galton in the 1880's (1883) and Spearman (1927) a little later, it has been widely assumed that the test measures “intelligence,” commonly referred to as “general cognitive ability,” or *g*. The identity of that ability, however has never been agreed; its function has only been characterized metaphorically as a kind of pervasive cognitive energy, power or capacity, by analogy with physical strength. In consequence, measuring it has always been indirect, creating perpetual debate and controversy about the validity of the tests. This article is about such validity.

## Validity of IQ Tests

In scientific method, generally, we accept external, observable, differences as a valid measure of an unseen function when we can mechanistically relate differences in one to differences in the other (e.g., height of a column of mercury and blood pressure; white cell count and internal infection; erythrocyte sedimentation rate (ESR) and internal levels of inflammation; breath alcohol and level of consumption). Such measures are valid because they rely on detailed, and widely accepted, theoretical models of the functions in question. There is no such theory for cognitive ability nor, therefore, of the true nature of individual differences in cognitive functions. A number of analyses of the inter-correlations of aspects of test scores have produced theories of the *statistical structure* of score patterns, as in the Cattell-Horn-Carroll theory (see McGrew, 2005); but this is not the same thing as detailed characterization of the function itself. Accordingly, as Deary (2001) put it, “There is no such thing as a theory of human intelligence differences—not in the way that grown-up sciences like physics or chemistry have theories” (p. ix).

The alternative strategy has been to attempt to establish test validity indirectly, by comparison of a proposed measure with what is considered to be some other expression of intelligence. Galton (1883) chose differences in social esteem; subsequently, scholastic performance and age-related differences were chosen. Typically, in constructing a test, cognitive problems or items thought to engage aspects of intelligence are devised for presentation to testees in trials. Those items on which differences in performance agree with differences in the criterion are put together to make up an intelligence test. There are many other technical aspects of test construction, but this remains the essential rationale. Thus, nearly all contemporary tests, such as the Stanford-Binet or the Woodcock-Johnson tests, rely on correlations of scores with those from other IQ or achievement tests as evidence of validity.

However, the question of whether such procedure measures the fundamental cognitive ability (or *g)* assumed has continued to haunt the field. Measuring what we think is being measured is known as the construct validity of the test—something that cannot, by definition, be measured indirectly. Generally, a test is valid for measuring a function if (a) the function exists and is well characterized; and (b) variations in the function demonstrably cause variation in the measurement outcomes. Validation research should be directed at the latter, not merely at the relation between what are, in effect, assumed to be independent tests of that function (Borsboom, Mellenberg, & van Heerden, 2005).

It is true to say that various attempts have been made to correlate test scores with some cortical/physiological measures in order to identify cerebral “efficiency” as the core of intelligence. However, as Nisbett et al. (2012), in their review for the American Psychological Association, point out, such studies have been inconsistent:

Patterns of activation in response to various fluid reasoning tasks are diverse, and brain regions activated in response to ostensibly similar types of reasoning (inductive, deductive) appear to be closely associated with task content and context. The evidence is not consistent with the view that there is a unitary reasoning neural substrate. (p. 145)

Haier et al. (2009) likewise conclude after similar inconsistent results that “identifying a ‘neuro-*g*’ will be difficult” (p. 136). Associations have also been sought between various elementary tasks such as reaction time and IQ test scores. These have been difficult to interpret because the correlations are (a) small (leaving considerable variance, as well as true causes, unexplained) and (b) subject to a variety of other factors such as anxiety, motivation, experience with equipment, and training or experience of various kinds such as video game playing (e.g., Green & Bavelier, 2012).

Accordingly, validation of IQ tests has continued to rely on correlation with other tests. That is, test validity has been forced to rely, not on calibration with known internal processes, but on correlation with other assumed expressions, or criteria, of intelligence. This is usually referred to as “predictive” or “criterion” validity. In almost all validity claims for IQ those criteria have been educational achievement, occupational level and job performance.

## Predictive Validity of IQ

It is undoubtedly true that moderate correlations between IQ and those criteria have been reported. For example, in their recent review Nisbett et al. (2012) say “the measurement of intelligence—which has been done primarily by IQ tests—has utilitarian value because it is a reasonably good predictor of grades at school, performance at work, and many other aspects of success in life” (p. 2). But how accurate and meaningful are such correlations?

It is widely accepted that test scores predict school achievement moderately well, with correlations of around 0.5 (Mackintosh, 2011). The problem lies in the possible self-fulfilment of this prediction because the measures are not independent. Rather they are merely different versions of the same test. Since the first test designers such as Binet, Terman, and others, test items have been devised, either with an eye on the kinds of knowledge and reasoning taught to, and required from, children in schools, or from an attempt to match an impression of the cognitive processes required in schools. This matching is an intuitively-, rather than a theoretically-guided, process, even with nonverbal items such as those in the Raven's Matrices. As Carpenter, Just, and Shell (1990) explained after examining John Raven's personal notes, “ … the description of the abilities that Raven intended to measure are primarily characteristics of the problems, not specifications of the requisite cognitive processes” (p. 408).

In other words, a correlation between IQ and school achievement may emerge because the test items demand the very kinds of (learned) linguistic and cognitive structures that are also the currency of schooling (Olson, 2005). As Thorndike and Hagen (1969) explained, “From the very way in which the tests were assembled [such correlation] could hardly be otherwise” (p. 325). Evidence for this is that correlations between IQ and school achievement tests tend to increase with age (Sternberg, Grigorenko, & Bundy, 2001). And this is why parental drive and encouragement with their children's school learning improves the children's IQ, as numerous results confirm (Nisbett, 2009; Nisbett et al., 2012).

Similar doubts arise around the use of occupational level, salary, and so on, as validatory criteria. Because school achievement is a strong determinant of level of entry to the job market, the frequently reported correlation (*r* ∼ 0.5) between IQ and occupational level, and, therefore, income, may also be, at least partly, self-fulfilling (Neisser et al., 1996). Again, the measures may not be independent.

The really critical issue, therefore, surrounds the question of whether IQ scores predict individual differences in the seemingly more independent measure of job performance. Indeed, correlation of IQ scores with job performance is regularly cited as underpinning the validity of IQ tests. Furnam (2008) probably reflects most views when he says that “there is a large and compelling literature showing that intelligence is a good predictor of both job performance and training proficiency at work” (p. 204). In another strong commentary, Kuncel and Hezlett (2010) refer to “this robust literature” as “facts” (p. 342). Ones, Viswesvaran, and Dilchert (2005) say that “Data are resoundingly clear: [measured cognitive ability] is the most powerful individual differences trait that predicts job performance … Not relying on it for personnel selection would have serious implications for productivity. There is no getting away from or wishing away this fact” (p. 450; see also Ones, Dilchert, & Viswesvaran, 2012). Drasgow (2012) describes the correlation as “incontrovertible.” Hunter and Schmidt (1983) even attached dollar value to it when they claimed that the U.S. economy (even then) would save $80 billion per year if job selection were to be universally based on IQ testing.

Unfortunately, nearly all authors merely offer uncritical citations of the primary sources in support of their statements (for exceptions see, for example, Wagner, 1994, and in the following sections). Instead of scrutiny of the true nature of the evidence, a conviction regarding a “large and compelling literature” seems to have developed from a relatively small number of meta-analyses over a cumulative trail of secondary citations (Furnham, 2008, p. 204). It seems important, therefore, to take a closer look at the quality of data and method behind the much-cited associations between IQ and job performance, and how they have been interpreted. The aim, here, is not to do an exhaustive review of such studies, nor to offer a sweeping critique of meta-analyses, which have many legitimate uses. Indeed, the approach devised by Schmidt and Hunter (1998), which we go on to discuss, brought a great deal of focus and discipline to the area and we agree with Guion (2011) that it must be recognized as a major methodological advance. Rather our aim is to emphasize the care needed in interpretation of correlations when based on corrections to original data of uncertain quality and then invoked as evidence of IQ test validity.

## Predicting Job Performance From IQ Scores

In contrast with the confidence found in secondary reports, even a cursory inspection of the primary sources shows that they are highly varied in terms of data quality and integrity, involving often-small samples and disparate measures usually obtained under difficult practical constraints in single companies or institutions. Their collective effect has mainly arisen from their combination in a few well-known meta-analyses. Hundreds of studies prior to the 1970s reported that correlations between IQ tests and job performance were low (approximately 0.2–0.3) and variable (reviewed by Ghiselli, 1973). These results were widely accepted as representative of the disparate contexts in which people actually work. Then, Schmidt and Hunter (2003, for an historical account) quite reasonably considered the possibility that the large quantity of results were attenuated by various statistical artifacts, including sampling error, unreliability of measuring instruments, and restriction of range. They devised methods for correcting these artifacts and incorporating the studies into meta-analyses. The corrections doubled the correlations to approximately 0.5. Nearly all studies cited in favor of IQ validity are either drawn from the Schmidt and Hunter meta-analyses or from others using the correction methods developed for them.

The Schmidt and Hunter approach (1998), as first devised, seemed relatively straightforward. First, the results were collated from as many studies as were available. Then, the variance due to sampling error in the reported (observed) correlations was estimated. Then, the mean of the observed correlations was computed and corrected for measurement unreliability in the criterion (i.e., job performance) and for restriction of range in predictor and criterion measures. This produced the results now so widely cited in vindication of IQ test validity (Hunter, Schmidt, & Jackson, 1982; Schmidt & Hunter, 1977, 1998).

Hunter and Hunter (1984) first reported the application of these methods—usually referred to as “validity generalization,” or VG—to the hundreds of studies reviewed by Ghiselli (1973). In addition, they reported a further meta-analysis of 515 studies carried out by the U.S. Employment Service using the General Aptitude Test Battery (GATB). This produced corrected correlations in the range 0.5–0.6. Similar results have been reported from application of the same methods in more recent studies. For example, in meta-analyses of European and British studies, Salgado et al. (2003) and Bertua, Anderson, and Salgado (2005) found raw correlations between 0.12 and 0.34, depending on job category. However, all correlations virtually doubled under correction. Lang, Kersting, Hülsheger, and Lang (2010) report similar results from meta-analysis of 50 studies in Germany.

## Doubts About These Studies

It is these corrected correlations from meta-analyses that are almost universally cited in favor of IQ as a predictor of job performance (and, by implication, that IQ really does measure something that can be called intelligence or general ability). But many doubts have been expressed regarding those methods, and results have been subject to continual criticism. Generally, meta-analyses are rarely straightforward and, at times, have been controversial. Although undoubtedly useful in many subject areas, as Murphy (2003) says, they are often viewed with distaste because they mix good and bad studies, and encourage the drawing of strong conclusions from often-weak data. In the IQ-job performance studies in question, quality checks are often difficult because the original reports were unpublished, sometimes with parts of original data lost. In addition, the corrections themselves involve many assumptions, for example about normality of distributions and randomness of effects, which are rarely articulated in primary reports (Murphy, 2003). Landy (2003) described them as the “psychometric equivalent of alchemy” (p. 157). The criticisms here will focus on both the quality of the primary data and the surety of the meta-analytic corrections to them. First, let us consider the measuring instruments used.

## The Many Surrogates of IQ Tests

However well-intentioned, most studies have been done under difficult circumstances so that study design, including choice of test, has often been based on convenience rather than principles of empirical precision. Accordingly, a wide variety of vaguely mental tests has been adopted across individual studies, and incorporated into meta-analyses, on the assumption that they measure essentially the same thing (by implication “general intelligence” or *g*). Apart from the traditional psychometrically validated instruments (e.g., Wechsler's Adult Intelligence Scale, Raven's Progressive Matrices, or the U.S. Employment Service's General Aptitude Test Battery), studies have included working memory tests, reading tests, scholastic aptitude tests (SATS) and university admission tests, all taken in meta-analyses as surrogate measures of IQ. Sometimes, a “general” factor has been deduced as a composite of “special ability” tests (e.g., perceptual speed, memory; Lang et al., 2010), or by renaming the construct “general mental ability” (GMA) as “another name for *g*” (James & Carretta, 2002, p. 13).

Illustrative of the variety of tests used in meta-analysis are those listed in the European study of Salgado et al. (2003). They include “(a) Batteries: DAT, GATB, T2, ASDIC, Intelligence Structure Test (IST-70), Wilde Intelligence Test (WIT), GVK, PMA, and Aptitudes Mentales Primarias (AMPE); (b) *g* tests: Raven's Progressive Matrices, Cattell's Culture Fair Tests, Otis Employment Test, Alpha Test, Logique Intelligence, CERP, Domino, D-48, NIIP-33” (Salgado et al., 2003, p. 1070). This categorization implies that “batteries” and “*g* tests” measure something different from each other—if so, what? More importantly, the studies using them cover a vast range of dates, some from the 1920s, while the majority are pre-1970s. These will not, of course, take any account of the “Flynn effect”—the substantial cross-generational rise in average IQ scores—which affects different tests differently and affects variances and distributions as well as means (Flynn, 2007; Wai & Ptallaz, 2011). Likewise, with the Bertua et al. (2005) meta-analysis of 60 UK studies: studies date from the 1920s to the 1980s, and utilized an equally wide range of disparate tests.

Further uncertainty is added by the high proportion of original studies involving men and women serving in the armed forces. These used a wide range of specialist and multi-purpose tests, such as the Armed Forces Qualification test, the Australian Army Intelligence Test, and the Armed Service Vocational Aptitude Battery. Sometimes, measures have been statistically reduced to a single component of variance, or primary factor, before meta-analysis (e.g., Olea & Ree, 1994). The usual justification for doing so is that any general factor condensed from inter-correlated scores can be assumed to represent *g*, and, therefore, that the tests are genuine tests of intelligence (even though a general factor typically covers only around 50% of the score variance). It is always a possibility, of course, that different correlates, even though resolving as a statistical “common factor,” may well not be the same “thing,” or even the thing it is thought to be, so that mischaracterization can occur. In the case of mental test performances, the general factor may not even be cognitive in origin (Richardson, 2002; see the following sections).

As Murphy (2003) says, the assumption that these measures, with disparate properties, distributions, and so on, can be combined as if a single uniform variable can lead to serious problems in meta-analysis including “lack of clarity in what population parameter is being estimated” (p. 31). Murphy and Newman (2003) add that, “if several hundred studies each claim to measure ability and performance, but they use wildly different measures of one or both constructs, the average ability-performance correlation across those studies might be hard to interpret” (p.414). Burke and Landis (2003) also complain about the “cavalier” treatment of construct issues in meta-analyses.

## Job Performance?

In contrast to the vast diversity of predictor tests, the measure of job performance has almost always consisted of supervisors’ ratings. These, of course, should be reliable, valid, and free from bias of whatever source. Unfortunately, as with ability testing, the strict requirements are often overlooked (Guion, 2006). It turns out that there are a number of problems with such ratings (Woerh, 2011).

The main problem is that supervisors tend to be subjective, and use inconsistent criteria, in making their judgments of performance. This is hardly surprising, given the difficulty of defining good or poor performance. As Gottfredson (1991) noted, “One need only ask a group of workers in the same job to suggest specific criterion measures for that job in order to appreciate how difficult it is to reach consensus about what constitutes good performance and how it can be measured fairly” (p. 76). In addition, a variety of systematic biases are evident: age effects and “halo” effects have been reported (e.g., Murphy & Balzer, 1986). Subjects’ height (Judge & Cable, 2004); facial attractiveness (Hosoda, Stone-Romero, & Coats, 2003); and unconscious ethnic bias (Berry, Clark, & McClure, 2011; Jencks, 1998; Stauffer & Buckley, 2005), have all been shown to influence supervisor ratings of work performance. In describing the difficulties, in his own experience, of seeking objective supervisor ratings across a wide range of jobs, Guion (2006) says, “Perhaps, indeed, we should abandon the pretence about ‘objective’, ‘true’, or ‘hard’ criteria of proficiency in performance” (pp. 268–269).

Perhaps it is hardly surprising, therefore, that supervisor ratings have rather low correlations with more objective criteria such as work samples or work output (Bommer, Johnson, Rich, Podsakoff, & Mackenzie 1995; Cook, 2009; Heneman, 1986). Schmidt, Hunter, and Outerbridge (1986) put it at virtually zero. In a study of salespersons, Vinchur, Schippmann, Switzer, and Roth (1998) found that “general cognitive ability” showed a correlation of .40 with supervisor ratings but only .04 with objective sales. Roth, Bobko, and McFarland (2005) found a mean observed correlation between work sample tests and measures of job performance (mostly supervisor ratings) of only 0.26, and a correlation between work sample tests and “general cognitive ability” of only 0.33. It is somewhat strange, therefore that Hunter (1986) reported that IQ/GMA predicted work sample ratings even better than it predicted supervisor ratings suggesting, perhaps, that they are measuring different things.

Another problem is the difficulty investigators have experienced in establishing reliabilities for supervisor ratings. Accurate reliabilities are needed, of course, in order to achieve the corrections to correlations. But they tend to be available for only a minority of the studies incorporated in the commonly cited meta-analyses. The strategy of Schmidt and Hunter and other meta-analysts has been to simply extrapolate from the average of those actually available. That strategy, of course, involves many assumptions about representativeness, randomness, uniformity across disparate samples, and so on. Using such a strategy, Hunter and Hunter (1984) assumed a reliability of 0.6 for their corrections, which some investigators have considered to be too low (Hartigan & Wigdor, 1989). Bertua et al. (2005) used the same figure for their meta-analysis of British studies. Moreover, that estimate was based on inter-rater reliability. Murphy and DeShon (2000) pointed out that differences between raters should not be considered error to be corrected because different raters may be looking for different things in a worker. Instead, *intra*-rater reliabilities should be used. However these tend to be much higher: 0.86 rather than 0.6. according to the meta-analysis carried out by Viswesvaran, Ones, and Schmidt (1996). The lower the value adopted, of course, the bigger the inflation to raw correlations. Using the reliability of 0.6, for example, inflates the correlations by 29%. By comparison, distinguished statistician John Hartigan, and colleague Alexandra Wigdor, favour the 0.8 estimate which only inflates the correlation by 12% (Hartigan & Wigdor, 1989) As Murphy (2003) says, evidence of error is so pervasive that many commentators urge caution in using supervisor ratings as criterion of job performance.

## The Corrections

In meta-analyses the reported correlation between IQ and job performance is a mean of observed correlations (usually weighted by sample size, if known). It could be that the low correlations from early studies are the true correlations for the general population of employees across their myriad jobs and contexts. Hunter and Schmidt (1977) argued, conversely, that the diverse correlations are artefacts of data collection. They devised a number of formulae for making corrections to them that have been refined over the years but remain essentially the same.

## Corrections for Sampling Error

First, sampling error arises because the observed (primary study) correlations are being estimated from sub-samples of the general population as well as sub-samples of the universe of jobs. The correlations, that is, will deviate from the (unknown) population correlation by an unknown degree, affecting the overall estimate as well as its confidence intervals. The mean of the observed correlations—as used in meta-analysis—will also have an inflated variance. Therefore, the sampling error variance has to be subtracted from the overall variance to arrive at the variance for the true correlation and it's statistical significance. Estimates for all these values need to be computed from the data. In using their methods and assumptions Schmidt and Hunter (1998) estimated that approximately 70% of the apparent variance consisted of sampling error variance.

A number of issues surround corrections for sampling errors. The Schmidt and Hunter approach (2003) assumes that all specific study samples are from essentially the same reference population with a single underlying IQ/job-performance correlation having close to zero variance. This assumption, together with the distribution of sampling errors, is used to indicate how close the average observed correlation is likely to be to the “true” correlation.

However, this maneuver is based on the further assumption that the primary studies are random samples from the (hypothetical) general population. This cannot be checked in samples where a number of details are missing. Rather than being carefully planned as random designs, particular studies are conducted on an as available basis, as Murphy (2003) puts it. After all, recruitment of participants is based on finding an employer willing to have employees tested and finding supervisors willing to rate them, which will be more likely to occur with some jobs than others. Hartigan and Wigdor (1989) provide evidence of such bias. Moreover, effects of systematic moderator variables are rarely taken into account (Schmitt, Gooding, Noe, & Kirsch, 1984). These can only be eliminated through primary research with appropriate controls (Russell & Gilliland, 1995).

*When* the corrections to sampling errors are done is also an issue. The estimated true mean correlation is computed as an average of observed correlations, as previously mentioned. Ideally, the sample means should be individually corrected for sampling error, measurement unreliability and range restriction *before* the averaging occurs; that is, meta-analysis should be done on the fully corrected samples. However, as most of that information is not available in the individual studies, the Schmidt and Hunter method (2003) corrects for them *after* the averaging, which can introduce further inaccuracies including reduction of observed variance and exaggerated sampling error variance (Davar, 2004; Oswald & McCloy, 2003). Hartigan and Wigdor (1989), in their meta-analysis of more recent studies, estimated sampling error to be about half the observed variability (compared with the 70% suggested by Schmidt & Hunter [1998]). In other studies (e.g., Burke & Landis, 2003; Lang et al., 2010) corrections have been based on the weighted mean of available estimates from other meta-analyses, or “hypothetical estimates” (Lang et al., p. 612).

## Corrections for Measurement Error

The sample means may also deviate from the hypothetical true mean because of unreliability of measurement, or measurement error, in both ability test and job performance assessment. A correlation between IQ and job performance in a specific study may be depressed because of such error. That also needs to be corrected. The main effect of correcting for measurement error is to increase the observed correlations usually in proportion to the unreliability of the measure: the greater the unreliability the bigger the upward correction to the correlation.

The desirable technique for measurement error correction consists of adjusting each coefficient included in the meta-analysis individually using reliability information provided for the specific predictor and criterion measures in the study report. In the most-used and reputable standardized tests reliability is well established and the attenuation can be corrected in advance of the meta-analysis. However, the reliabilities of the measures actually used in the meta-analyses in question were “only sporadically available” (Hunter & Schmidt, 1990, p. 79). They recommended basing them on the subset of the studies for which information happened to be available.

Using that strategy, Schmidt and Hunter (1977) arrived at a reliability of .60 for job performance. As Hartigan and Wigdor (1989) explained, this figure “has met with some scepticism among industrial/organizational psychologists many of whom believe that the .60 value is too low” (p. 166). The overall effect of using the .60 value is to increase the estimate of the population correlation by 30%. This too has remained an area of controversy (Sackett, 2003).

More generally, although correcting for measurement error seems straightforward and desirable, it is theoretically more complicated and may not always be consistent with psychometric principles (Murphy & DeShon, 2000). DeShon (2003) says, “there are numerous theoretical reasons for urging caution when correcting the magnitude of the correlation coefficients for measurement error”, and it “is of dubious merit in many situations” (p. 382). One of these is that, although correcting for measurement error will often increase the correlation coefficient, it also increases its standard error with larger confidence intervals not differentiating it from zero. Reliabilities of job performance ratings are computed from estimates on different occasions. However, differences in estimates may be due to genuine differences in performance rather than measurement error. Most individuals create a difference between their maximum and their typical performances such that these indices are not highly correlated and have different correlates (Marcus, Goffin, Johntson, & Rothstein, 2007). Stewart and Nandkeolyar (2006) found that intra-individual variation was greater than *inter*-individual variation in job performance. Again, correction becomes, to some extent, guesswork, yet the adjusted correlations depend upon it.

The statistical model used for meta-analysis and its corrections may also be an issue here. Correction of measurement error is based on a random effects model, but the unreliability of (in this case) supervisor ratings may stem in part from a number of systematic (i.e., non-random) effects (Murphy & DeShon, 2000). For example, different job contexts may involve different kinds of disagreement among raters about what should be measured or about how the rating scales should be used. Also, there may be systematic differences among testees related to, for example, gender, ethnic background and social class background and the effects of these on such variables as self-confidence and ability expression (see subsequent sections). A variety of studies indicate that “macrosocial differences in the distribution of economic goods are linked to microsocial processes of perceiving the self” (Loughnan et al., 2011, p. 1254). Such perceptions can impinge on correlations between test and job performances. These are non-random errors that complicate inferences from particular samples used in particular times and places (DeShon, 2003).

Correcting for measurement error also has complex effects on the variances of the observed correlation coefficients. As implied above, corrections for measurement error made after, rather than before, averaging in meta-analyses, may exaggerate sampling error variance and reduces the variance of the estimated correlation. Much more statistical evaluation of the combination of known and unknown measurement unreliabilities is called for “before this procedure could be recommended as general practice” (DeShon, 2003, p. 397).

More generally, measurement error may also arise on account of the lack of construct validity (the proof that it is measuring the function intended). It is, of course, the acknowledged lack of construct validity in IQ testing that has led to such reliance on predictive validity in the first place. Lack of it, nevertheless, has implications for corrections for unreliability in meta-analyses. Schmidt and Hunter's approach (1977) insists that correcting for measurement error provide an estimate of the “true” correlation between the underlying constructs. Borsboom and Mellenbergh (2002), on the basis of classical test theory, have vehemently disagreed with this because it also assumes what it is trying to prove, namely the validity of that construct being revealed through the test-criterion correlation. As Burke and Landis (2003) explain:

Meta-analytic research … is sometimes cavalier in its treatment of construct-related issues. In particular, there sometimes is an apparent assumption that superficially similar studies, or those that claim to be dealing with the same set of constructs, can be easily combined to draw meaningful construct-level inferences. This is not true. Rather, careful thought needs to go into decisions about how to link study outcomes with constructs. (p. 298)

## Corrections for Range Restriction

The third common problem is that sample correlations may vary because of range restriction in the samples, compared with the general population. The main reason it arises is that job performance ratings can only be provided for those who are actually in the job, and have been IQ tested, not for all *possible* workers (including applicants who did not get the job). An unmeasured complication is that those who might even apply for a job will be self-selecting to some extent, reflecting self-perceptions of a variety of other attributes such as experience, ability, self-confidence, experience, paper qualifications, and so on. The statistic needed to correct for range restriction is the ratio of the observed standard deviation (*SD*) in the restricted sample to that in the unrestricted population. For example, if the ratio is 0.5 the effect of correction is to double the sample correlation. Legitimate correction depends, of course, on having accurate estimates of *both* sample and population variances. As with measurement unreliabilities, however, few primary studies have reported range restrictions, so that extrapolation is again necessary, and again with all the dangers entailed.

The main problem is that of identifying the variance for the appropriate reference population. In the present case the true reference population is all applicants for a job—all of which should have been IQ tested—from which a limited proportion are recruited for the job and assessed for job performance. However, the standard deviation (*SD*) of the observed (job applicants’) test results is rarely available. So the strategy has been to deduce it from that of actual workers’ scores, the only ones available. In the Schmidt and Hunter methods it is simply assumed that the reference population *SD* could be represented by the “entire US workforce” which could, in turn, be adequately represented by the 515 jobs in the (then) GATB database. The *SD*s for those samples available were then compared with this overall SD as the basis for correction of range restriction for all the samples. Schmidt and Hunter (1977) thus arrived at a restriction ratio of 0.6.

The review of these studies by Hartigan and Wigdor (1989, p. 167) says that the assumption that the applicant pool for each and every job can be approximated by the GATB workforce is “troubling.” As previously mentioned, it is rarely clear to what degree a particular sample may be restricted, in relation to the reference, because people tend to be self-sorting in the jobs they seek rather than belonging to a random applicant pool. In other words, it is likely that employee samples will display inhomogeneity, and not be representative of normative data (Lang et al., 2010). This inhomogeneity is more likely with smaller samples. Hunter and Hunter (1984) cite earlier studies as having average sample sizes of just 68, which means some must have been even smaller than that. Schmidt and Hunter (1998) say that *n*'s were usually in the 40–70 range. This is also important in that there are certain situations, such as non-normal data with outliers, in which the correction can actually decrease rather than increase the correlation (Zimmerman & Williams, 2000).

In sum, there is a danger that adjustments for any of these parameters will over-correct, making the validity coefficients spuriously large (Wagner, 1994). As Hartigan and Wigdor (1989) stress, the device of using an average figure for population variance could lead to inflated corrections for restriction of range, and argue that, in the absence of clear information for each group, the safest thing is to apply no corrections.

As it is, Schmidt and Hunter's (1998) corrections inflate the correlations in their samples by 61% when combined with their correction for measurement unreliability. Hartigan and Wigdor's (1998) own estimates increased the correlation by only 12%, to 0.22, compared with Schmidt and Hunter's (1998) 0.51. Their critique has been taken up by other critical reviews in, for example, Cook (2009), McDaniel (2007), and Jencks (1998), reiterating their cautionary notes. There have been attempts to refine these correction methods (e.g., Le & Schmidt, 2006), albeit with further assumptions and approximations for missing data, and, therefore, the debate continues.

## Summary of Doubts About Corrected Correlations

It needs to be emphasized, again, that the meta-analytic approach used in this area has been generally well accepted and even critics tend to urge cautions and further questions rather than complete dismissals. We now review these, try to add a few more, and stress the dangers of drawing strong conclusions. As Murphy (2003) says the “long and bitter controversy” over the use of these corrections in validity studies is partly due to the way that strong claims have been made from mixed primary data. Pointing to a number of statistical issues, Bobko and Roth (2003) similarly suggest that proponents of meta-analysis “may be a bit over-zealous in claims about what meta-analysis could or could not accomplish’, and that “caveats … are in order” (p. 68). The main problems stem from weaknesses and uncertainties in the primary data. Schmitt, Arnold, and Nieminen (2010) suggest boldly that the absence of data in most primary studies simply does not allow “for sample-based corrections for criterion unreliability or range restriction” (p. 66). Kaufman and Lichtenberger (2006, p. 18) also warn against “incautious and, perhaps, overzealous corrections” of primary correlations.

Moreover, biases may have arisen from the fact that statistically significant findings, or ones that conform to previous results, are more likely to have been published than nonsignificant, or low effect, findings (known as the “file-drawer” problem; Field, 2007; Murphy, 2003). McDaniel, Rothstein, and Whetzel (2006) analyzed the validity claims in the technical manuals of four test providers that used supervisor ratings as criterion. They noted that two of the publishers tended to report only statistically significant correlations. We can only guess the extent to which this problem has affected results of meta-analyses.

More important, perhaps, is the problem of how to interpret the corrected correlations. Most uncritical readers have accepted corrected correlations as the “true” correlations. It is probably more prudent, however, to interpret them as theoretical maximum correlations *given* such weak samples and unreliable test instruments: “elevated idealized correlations rather than actual correlations” (Sternberg et al., 2001, p. 10). Or, as Kaufman and Lichtenberger (2006) put it, “these corrections inflate the correlations by estimating their magnitudes in ‘what-if’ situations” (p. 18); for example, what the correlation might be in ideal conditions with perfectly reliable testing instruments, which do not exist.

Finally, nearly all studies are concurrent in design: instead of testing predictor at one age/time and then the criterion some time later the measures of both are usually taken more or less together. As Banks and McDaniel (2014) discovered, this may overestimate the validity “perhaps substantially.”

Note that similar claims have been made about correlations between IQ and *training* success in various occupations. Schmidt and Hunter (1998) indicate a correlation of 0.54, and that figure has been widely accepted (Bertua et al., 2005; James & Carretta, 2002). But they are subject to the same objections as those for job performance: the raw correlations are very low (around 0.2), doubled or more in the meta-analyses through estimated corrections. The most quoted results are from training in Forces personnel, whereas all meta-analyses include dozens of different tests, of varying psychometric standards, and many very old studies, dating as far back as the 1920s (e.g., Bertua et al., 2005).

## More Recent Studies

As mentioned already, most of the studies incorporated into meta-analyses, from which the corrected correlations are widely cited, are pre-1970s. Some of the issues arising are illustrated in the report, already previously mentioned, by Hartigan and Wigdor (1989). This is the report of a Committee set up by the U.S. National Academy of Science to consider whether the U.S. Employment Service might promote the use of the GABT routinely throughout the country. Though broadly supportive, the committee's report critically commented on all the corrections reported in Hunter and Hunter (1984), based on the GATB, especially those based on assumptions not supported by available data.

As stated in Hartigan and Wigdor (1989), the 515 studies of Hunter and Hunter (1984) were conducted in the period 1945–1970: 10% in the 1940s; 40% each in the 1950s and 60 s; and 10% in the 1970s. However, a further 264 studies around the GATB were conducted after that and analyzed in the same report. As Hartigan and Wigdor (1989) note “The most striking finding … is a distinct diminution of validities in the newer, post 1972 set” (p. 150). These are described as “puzzling and obviously somewhat worrisome” (p. 160), and, therefore, other factors were considered. For example, the 264 newer studies have much larger average sample sizes (146 c.w. 75). It was shown how the larger samples produced much lower sampling error, requiring less correction. They also produced much lower variation with job family (or level of job complexity, see the following section). The more recent studies also exhibited less range restriction, also requiring less correction (with much less possibility of a false boost to observed correlations). These findings were supported by Jencks (1998) who noted that “GATB scores do not predict job performance very well”, and that “for reasons nobody understands, the GATB's ability to predict job performance has been falling” (p. 75).

Another explanation for the lower IQ/job performance correlations in the more recent years may lie in the general skill-upgrading of jobs, with reduced differences in the cognitive demands of occupations. This is, of course, also an explanation sometimes offered for the so-called “Flynn effect” concerning the substantial rise of average IQ scores over time (Flynn, 2007). The effects of reduced variance of output together with rising “inputs” may be inter-twined such as to also reduce the IQ/job-performance correlation over time. In addition, it may be that higher IQ test performance *and* more favorable job supervisor ratings *both* reflect a variety of mediating non-cognitive factors such as self-confidence (see further the following section).

Finally, it seems that even the weak IQ-job performance correlations usually reported in the United States and Europe are not universal. For example, Byington and Felps (2010) found that IQ correlations with job performance are “substantially weaker” in other parts of the world, including China and the Middle East, where performances in school and work are more attributed to motivation and effort than cognitive ability.

## The Job Complexity Issue

Based on their meta-analyses of studies using the GATB, Hunter and Hunter (1984) categorized jobs based on impressions of the complexity of cognition demanded. They claimed that the correlation between IQ and job performance is stronger in the more complex jobs. Much has been made of that claim in the many subsequent citations of it. Thus, Gottfredson (1997) said that, “An especially important observation is that predictive validities vary systematically according to the overall complexity of the work involved” (p. 82). On the basis of the same meta-analyses, Ones et al. (2012, p. 189) reiterated that “relationships … are strongest for highly complex jobs (e.g., attornies, medical doctors, pilots). The validities in medium complex jobs are somewhat lower … (mostly in 0.50 s). Even for low-complexity jobs, criterion correlations are in the useful range (0.20 s)”. But how true is this inference? Is this what the data unequivocally show?

First of all, as already mentioned, the meta-analyses include studies that are very old with much missing data. The association may itself be an artefact of “corrections for artefacts” in such compromised empirical circumstances. Table 1 compares those correlations with others from the newer studies reported by Hartigan and Wigdor (1989). Although following a similar correction protocol as Hunter and Hunter, the newer correlations are remarkably uniform (and small) across all job complexity categories. When Hartigan and Wigdor corrected the newer 264 studies for only sampling error (because they were suspicious of the empirical justification for other corrections) the correlations were very low (0.06–0.07) and virtually identical across job families.

**TABLE 1:** Correlations From Older and More Recent Meta-Analyses

*Level*	*Hunter Studies*		*Newer Studies*	
	*UC*	*C*	*UC*	*C*
I	0.31	.56	0.15	.17
II	0.14	.23	0.19	.21
III	0.30	.58	0.25	.28
IV	0.27	.51	0.21	.23
V	0.20	.40	0.18	.20

*Note.* UC = uncorrected; C = corrected.

Data from Hunter and Hunter (1984) and Hartigan and Wigdor (1989). In Hunter's two classification schemes I is “precision setup” group (e.g., machinist, cabinetmaker, metal fabricator); II is feeding-offbearing group (e.g., shrimp picker, corn-husking machine operator, cannery worker); III is “high complexity” (e.g., retail food manager, fish and game warden, biologist, city circulation manager); IV is “medium complexity” (e.g., automotive mechanic, radiologic technician, automotive parts counterman, high school teacher); V is “low complexity” (e.g., assembler, insulating machine operator, forklift truck operator).

Where the correlations do vary, however slightly, that may be attributed to other systematic effects across job categories. As previously noted, people do not, generally, perform as well as they could in most situations and supervisor ratings are likely to report typical rather than maximal performances, perhaps depending on working conditions. More complex jobs will usually offer more congenial working conditions and more equal relationships between managers and employees (indeed, many of them will *be* managers), thus ameliorating many of the psycho-situational variables such as stress and anxiety that can interfere with both test performance and job performance (see the following section). That is, workers are more likely to perform asymptotically in more congenial (i.e., higher class) jobs than in less congenial jobs, boosting the correlation between IQ and job performance. Jobs of different complexity will also vary systematically with other psychological attributes of testees and job situations. Testees are from distinct social class backgrounds associated with different levels of preparedness for both test and job performance. For example, higher-class jobs will usually be associated with important psychological attributes of testees, such as abundant self-esteem and self-efficacy beliefs (Bandura, 1997; Dweck, 2008). At those levels, testees are more likely to be from the same social class as their performance raters (with the bias effects described earlier). Conversely, it is observed that those in lower complexity/lower class jobs are likely to have less frequent and less skillful communication with supervisors (Guion, 1983).

## Of Correlations and (Non-Cognitive) Causes

Perhaps the biggest problem throughout this validation history has been the readiness with which correlations have been accepted as causes: that is, the inference that individual differences in IQ test performances really are differences in a general mental ability because they are associated with individual differences in job performance. Correlations are repeatedly described in terms of “the effects” (of whatever an IQ test measures on job performance), instead of mere statistical covariation that does not, of itself, reveal the source(s) of that covariation.

That the causes may be more complex than a unitary cognitive factor is indicated by a number of anomalies in the findings. Further analyses of inter-correlations between factors surrounding correlations between IQ and job performance (i.e., path analyses) have led to the suggestion that any causal effect of cognitive ability on job performance is indeed indirect. For example, Schmidt, Hunter, and Outerbridge (1986) found that supervisor ratings had virtually zero correlations with actual samples of work performance, as previously mentioned. However, they exhibited a correlation of 0.3 with subjects’ job knowledge. In an experimental study, using regression analyses, Palumbo et al. (2005) found that cognitive ability accounted for 12% of variance in performance, but this was completely mediated by the association between cognitive ability and job knowledge. They thus recommend replacing IQ tests with job knowledge, or job understanding, tests as better predictors of job performance.

As Wagner (1994) says, disentangling causal effects from these associations requires additional constructs. It could be, as Schmidt and Hunter (2004) argue, that “general mental ability” (GMA) is related to job performance because it determines speed of acquisition of job knowledge, as well as its complexity; that is, simply another expression of *g*. But, however plausible that argument, it means accepting that an already small (∼0.3) correlation between job performance and performance on a pencil and paper test of job knowledge is entirely determined by an uncharacterized construct (*g*), the test of which is still lacking in acceptable construct validity. This is what led Wagner to complain that “we appear to have been blinded by what we have termed the ‘g-eocentric’ view” (p. 137).

The danger is that of viewing job knowledge as, itself, a pure variable, when its acquisition is probably affected by a range of other variables, known and unknown. For example, individual job knowledge is likely to be a function of prior experience, irrespective of level of the hypothetical *g*, and degree of experience can influence both IQ test performance and supervisor ratings of performance. Indeed, organizations tend to look carefully at previous experience in selecting candidates for a job. Research suggests that prior experience, as expected, tends to have a positive effect on job performance; however, it can also, in some individuals, have a negative effect on performance via behavioral and cognitive rigidity (Dokko, Wilk, & Rothbard, 2008). There is, of course, much evidence that IQ test performance can be boosted by—presumably knowledge-based—experience with compatible cognitive tasks (e.g., Mackey, Hill, Stone, & Bunge, 2011; Moreno et al., 2011).

It is because of such doubts that alternative, or additional, causal pathways in the correlations between IQ (or job knowledge) and job performance have been explored. The possible role of motivation was mentioned above. But other affective and contextual factors have been considered in recent years. In his studies, *Working with Emotional Intelligence,* Goleman (2000) found that *“67 percent -* two out of three - of the abilities deemed essential for effective performance were emotional competencies. Compared to IQ and expertise, emotional competence mattered twice as much. This held true across all categories of jobs, and in all kinds of organizations” (p. 31) (however, see Landy, 2005, for difficulties of testing).

According to Arthur and Villado (2008), the focus of personnel selection research is increasingly taking the “applicant perspective,” including “applicant reactions to selection systems, processes, methods, and decisions and the relationships of these reactions to outcomes, such as perceptions of fairness, face validity, test-taking motivation, test performance, and self-withdrawal from the selection process” (p. 435). These, too, may vary systematically, as previously noted. Similarly, the importance of work context on performance, as a crucial source of variance, has recently been studied, and shows the relationship between apparent ability and job performance to be remarkably labile. For example, Groysberg (2010), after examining the careers of more than a 1000 high performers (“star” analysts) on Wall Street, showed that those who change firms tend to suffer an immediate and lasting decline in performance. Performance seems to have depended more on their former firms’ organizational support, networks, and colleagues than the intellectual attributes of the individuals. This may partly explain why even the weak IQ-job performance correlations reported do not pertain outside of the United States and Europe, as previously mentioned.

Other factors can depress performance in both IQ tests and jobs below true ability. For social structural reasons, low-income parents “face a tax on their psychic resources” (Mullainathan, 2012). Testees/employees overwhelmed with worries about rent, feeding and clothing children, paying household bills, and reduced sense of control over circumstances, can suffer from a reduced “mental bandwidth” equivalent to a 13-point loss in IQ test performance (Mullainathan). They will also tend to have reduced motivation and self-confidence, and increased anxiety in both test and work situations. Ackerman and Heggestad (1997) reported a correlation of *r* = −0.33 between test anxiety and performance. Raven, Raven, and Court (1993, p. G14) note how fatigue, ill health and stress affect speed and accuracy on the RPM. In a meta-analysis Duckworth, Quinn, Lynamc, Loeberd, and Stouthamer-Loeberd (2010) showed that, after adjusting for test motivation the predictive validity of intelligence for life outcomes was significantly diminished, particularly for nonacademic out-comes. This means that those study participants will tend to perform below their best, or more erratically, on both predictor and criterion measures, thus lowering the correlation between them. Such considerations ought at least to moderate the strong claims usually made about the predictive validity of IQ tests drawn from correlations with job performance.

## DISCUSSION

We have urged caution in using IQ-job performance correlations for supporting the validity of IQ tests. The vast bulk of that reliance is based on the results of meta-analyses combining studies of variable quality involving corrections and estimates that many have criticized. However, meta-analyses are generally well-respected techniques with many supporters. It may, therefore, be appropriate to consider the specific contentious topics arising within the context of this particular article (for wider discussion of “matters still at issue” see Guion, 2011).

### The Diversity of Tests: Does It Matter?

Primary studies have often chosen the most convenient rather than the most appropriate tests, from simple reading or memory tests to the highly respected Raven. This diversity has been viewed in two ways. On the one hand, is the view that we cannot be sure what is being measured with such a variety of tests and with what psychometric properties, especially when combined in meta-analyses. On the other hand is the view that the emergence of significant predictive correlations, across a wide variety of tests demonstrates the robustness of the effect and, therefore, of conclusions from it.

Of course, it has to be remembered that many if not most of the primary studies report small and/or non-significant correlations, anyway: it is only their corrected composites in meta-analyses that can be called robust. In our view, there are two answers to the question. On the assumption of genuine and substantial correlations it can be said that the diversity of tests does not really matter, as long as the aim is mere prediction (after all a vast variety of other non-psychometric indices of job performance exist, including track record, interest inventories, language dialect, self-presentation, and so on). It certainly does matter, however, if the correlations are to be used for theoretical explanations of what is actually creating individual differences, as in developmental or career selection purposes or expensive genetic association studies—or for justifying the validity of IQ tests as measuring what we believe them to measure.

Such justification is based on the claim that, because scores on different tests inter-correlate to some extent, each test, however specialized, is also a measure of a general factor, *g*. Schmitt and Fandre (2008) suggest that “all are equally representative of a general factor *g*” (p. 169). The psychological identity of that factor is, however, another matter. The inter-correlation between tests may be due to something different from what we think it is, especially when there is so much disagreement about the identity of *g* and human intelligence.

### Identity of the Predictor Variable: *g* or Not *g*?

Almost any glance at the literature confirms the level of such disagreements. Take, for example, recent contributions to the *Cambridge Handbook of Intelligence* (2011; see Sternberg & Kaufman, 2011). Davidson and Kemp (2011) note that “Few constructs are as mysterious and controversial as human intelligence”, and that “there is little consensus on what exactly it means … for one person to be more intelligent than another” (p. 59). They also suggest that this heterogeneity of views has increased in recent times. Urbina (2011) reviews some of the “excessive and unjustified meanings that the IQ label has acquired” (p. 22). Sternberg and Kaufman (2011) simply say “there has never been much agreement on what intelligence is” (p. xv).

Charles Spearman, the originator of the term *g*, believed that it reflected differences in ability for the “eduction of relations and correlates” (Spearman, 1927). Schmidt and Hunter (2003) define it as learning ability. This is consistent with Gottfredson's (2007) view that *g* reflects differences in “general capacity to learn and reason” and that “all mental tests measure mostly *g*, whatever their content” (para. 2). Mackintosh (2004) on the other hand, reminds us that “*g* reflects no more (and no less) than the indisputable fact that scores on all IQ tests are positively correlated. Equally indisputably, however, we have little idea of the reason(s) for this positive manifold” (p. 217).

The problem with the “*g* is learning ability” argument is that it cannot be measured independently of an instrument that requires rather *specific* learning that is or has been more available to some social classes or (sub-) cultures than others. Trying to distinguish the “learned” from the “learning potential” is impossible (Kaufman & Lichtenberger, 2006). Simply introducing nonverbal items is not enough. Indeed, analysis of the content of items like those in the Raven (supposedly the most *g*-loaded test) suggests that they are the *most*, not the least, dependent on specific learning (Richardson & Norgate, In press). Because specific cultural tools (language, work, technologies, cultural methods, and practices) are the medium of all human transaction and learning, the very idea of a culture-free test is “a contradiction in terms … by its very nature, IQ testing is culture bound” (Cole, 1999, p. 646).

In other words, testees can be more or less prepared for the test by having acquired knowledge and cognitive styles in cultural formats more or less distant from the specific format of most tests. We cannot distinguish cognitive “strength” from cognitive “distance.” Additionally, there is abundant evidence of ability for cognitive activity much more complex than that in IQ test items, verbal or nonverbal, in everyday activities of most people (Richardson & Norgate, In press).

### Identity of the Criterion Variable

Job performance may seem, superficially, to be a perfectly unambiguous and stable criterion of intelligence. More recent research has shown that notion to be too simple: job performance is a much more complex entity that varies with a host of tangible and intangible factors. According to Sackett and Lievens (2008) the recent trend is an emerging new view of job performance beyond a single unitary concept to a more differentiated model. We noted earlier the suggestion of Guion (2006, pp. 268–269) for abandoning the pretense about objective or “hard” criteria of proficiency in performance. This is one reason why simple ratings, as in nearly all the IQ-job performance literature, need to be treated with skepticism.

### Status of Meta-Analysis

As already mentioned, our concern is not meta-analysis *per se*, which, together with the innovations of Schmidt and Hunter, have become respected techniques, but with its more narrow application to IQ test validity. We simply draw attention to problems surrounding the quality of primary data, the legitimacy of corrections, and the strength of conclusions drawn from them, urging caution about questions where high precision is needed. The main issue surrounding the Schmidt and Hunter approach (1998; the main source of alleged IQ test validity) is the validity of the corrections. A number of those were previously mentioned in this article. Here, we can only emphasize how even strong supporters demur. McDaniel (2007) constructively reviews the many detailed demands of an adequate meta-analysis. It is clear that they are not fully met in the case of IQ and job performance. Banks and McDaniel (2014) note that data analysis techniques cannot overcome poor reporting practices in primary studies. Guion (2011) expresses serious doubts about using very old primary studies (which he refers to as “rancid data”). He emphasizes that validities (IQ-job performance correlations) can change over time; and suggests that “the early computer warning (garbage in, garbage out) seems equally applicable to meta-analysis” (p. 265). Sackett (2003) notes continuing controversy about the appropriate use of some reliability estimates in meta-analytic corrections. In spite of increased confidence with meta-analysis, generally, as Schmitt and Fandre (2008) say, it is obvious that “important gaps in our understanding of ability-performance relationships remain” (p. 167). Strangely, Schmidt and Hunter (1998) did not respond to the fundamental critique of Borsboom and Mellenbergh (2002) which has attracted much support in the literature. Humphreys (1986), perhaps, put it more simply: “Given the heterogeneity among the many studies to be aggregated, corrections … are rough estimates at best” (p. 427).

### A “Large and Compelling Literature”

The impression of Furnham (previously cited) of “a large and compelling literature,” reporting essentially the same finding, is widely shared among readers of secondary reports. As a number of commentators have noted, at least some of the impression has been created by the fact that “Proponents of validity generalization have not been shy about making sweeping claims about the implications of their findings” (Murphy & Newman, 2003, pp. 417–418). It may be unfortunate that such over-zealousness appears to have been carried over into IQ advocacy by psychologists.

The reality is of a handful of meta-analyses pooling hundreds of studies of variable quality (many very old, with missing data, and so on) corrected with many assumptions and estimates. A multiplicity of studies of variable standard is no substitute for properly conducted primary studies, with larger representative samples, clearer measures, and so on. Until they are done, we suggest the validity of IQ tests remains an open question, especially when there are alternative explanations.

### Alternative Explanations

So what else could the correlations (such as they are), and the “positive manifold” among test performances be attributable to? One possibility is that both IQ and job performance reflect specific culturally-related learning, or cognitive preparedness, as already mentioned. Another is that the correlations could be entirely or partially non-cognitive in origin. Remember that a correlation is simply a measure of covariation of scores/ratings as reflected in degrees of deviation from respective means, without identifying the source of the covariation. Even covariations that are slight in relation to the respective measurement ranges can yield substantial correlations.

Many non-cognitive factors are known to jointly influence test performance and job performance such as to possibly yield such correlations. Levels of self-confidence, stress, motivation, and anxiety, and general physical and mental vigor, all affect cognitive test and job performances that will, therefore, tend to correlate (Derakshan & Eysenck, 2009; Dweck, 2008; Richardson & Norgate, In press). In addition, “macrosocial differences in the distribution of economic goods are linked to microsocial processes of perceiving the self” (Loughnan et al., 2011, p. 1254).

Turning the usual argument on its head, we suggest that inter-correlation of scores among such a diversity of tests actually *suggests* common noncognitive factors in operation. In other words, the “general factor” is (at least partly) an affective rather than a cognitive one. Factors of cognitive and affective preparedness could also explain the enigmatic Flynn effect (of rise of average IQ scores across generations), which cannot be explained by a general cognitive factor (Nisbett et al., 2012). However, the effect is readily explained by the demographic swelling of the middle classes in developing societies and the joint effects of better cognitive and affective preparedness (self-confidence, motivation, etc.).

### So What Do We Get?

To supporters of IQ testing (as cited earlier) the picture seems crystal clear. Job performance must be a good test of individual differences in intelligence. IQ test scores (or their surrogates) correlate significantly with ratings of job performance. As a result, IQ tests must be a valid test of intelligence.

What we actually have are scores from a predictor of nebulous identity correlated with ratings for a seemingly discrete construct that is turning out to be equally slippery. In other words, very strong conclusions are seemingly being drawn from correlations between two under-specified constructs. This makes interpretation of the (modest) correlations extremely difficult. In primary studies such correlations have generally left over 95% of the variance unexplained (Kaufman & Lichtenberger, 2006). Even the typical meta-analytic correlation of 0.5 still leaves 75% of the variance unexplained. This does not seem to us to constitute grounds for asserting test validation so strongly.

## SUMMARY AND CONCLUSION

Supporters of IQ testing have been quick to point to correlations between IQ and job performance as evidence of test validity. A closer look at the data and results, however, suggests a rather murkier picture. Here we have acknowledged the methodological advances in meta-analyses from which such evidence has been drawn, while drawing attention to the problems surrounding them in this particular area. We conclude with a summary of the main points:

1. Much in developmental theory, and psychology in general, depends upon the validity of IQ tests.
2. In the absence of agreed construct validity this has weighed heavily on indirect validity using correlations with criterion outcomes among which job performance has a special status.
3. Hundreds of studies prior to the 1970s reported low and/or inconsistent correlations between IQ and job performance.
4. These correlations have been approximately doubled using corrections for supposed errors in primary results and combining them in meta-analyses. Such corrections have many strengths, theoretically, but are compromised in these cases by the often uncertain quality of the primary studies.
5. The corrections to sampling errors, measurement errors, and to range restriction have required making a number of assumptions that may not be valid and have created a number of persistently contentious issues.
6. The claim that the IQ-job performance correlation increases with job complexity is not born out in more recent studies.
7. A range of other—including noncognitive—factors could explain a correlation between IQ and job performance, and even constitute part or all of the enigmatic “general factor.”
8. There remains great uncertainty about the interpretation of IQ-job performance correlations and great caution needs to be exercised in using them as a basis for the validity of IQ tests and associated concepts.

As others have pointed out, statistical corrections are no magical compensation for weak data and that it is risky to reach conclusions about test validities from those currently available (Oswald & McCloy, 2003; Russell & Gilliland, 1995). The only solution is properly conducted primary studies, with larger representative samples, better measures, and so on. Until they are available, investigators should be extremely cautious about disseminating conclusions about IQ test validities, from correlations between IQ and job performance.
